# Biomarker response to balloon-in-basket pulsed field ablation: does posterior wall isolation matter?

**DOI:** 10.3389/fcvm.2026.1733642

**Published:** 2026-03-03

**Authors:** Sascha Hatahet, Sorin Popescu, Charlotte Eitel, Suzanne de Waha, Tanja Zeller, Karl-Heinz Kuck, Jan-Per Wenzel, Roland Richard Tilz

**Affiliations:** 1 Department of Rhythmology, Heart Center Lübeck, University Hospital Schleswig-Holstein, Lübeck, Germany; 2 German Center for Cardiovascular Research (DZHK), Partner Site, Lübeck, Germany; 3 Institute for Cardiogenetics, University Hospital Schleswig-Holstein, Lübeck, Germany

**Keywords:** atrial fibrillation, hemolysis, inflammation, myocardial injury, PFA, platelets, posterior wall isolation, PVI

## Abstract

**Background:**

A novel balloon-in-basket pulsed field ablation (BiB-PFA) catheter enables efficient pulmonary vein isolation (PVI) and allows posterior wall isolation (PWI) within the same procedure. The incremental biological effect of PWI compared to PVI alone remains uncertain, particularly regarding inflammation, myocardial injury, and hemolysis.

**Methods:**

In this prospective, single-center study, consecutive patients with atrial fibrillation underwent first-time BiB-PFA, either PVI only or PVI plus PWI. Venous blood samples were collected before and one day after ablation. Biomarkers included leukocytes, platelets, hemoglobin, C-reactive protein (CRP), haptoglobin, bilirubin, lactate dehydrogenase (LDH), creatinine, estimated glomerular filtration rate (GFR), myoglobin, creatine kinase (CK), and troponin T.

**Results:**

A total of 60 patients were enrolled (PVI only *n* = 30, PVI + PWI *n* = 30). Baseline characteristics were comparable. PVI + PWI required more applications (19 vs. 16; *p* < 0.001) but had similar procedure time. Both groups showed significant increases in inflammatory (CRP, leukocytes), myocardial (troponin T, CK, LDH, myoglobin), and hemolysis markers (bilirubin, LDH, haptoglobin changes; all *p* < 0.001). However, the magnitude of biomarker release did not differ between PVI only and PVI + PWI: Δ troponin T (1,154 vs. 1,029 ng/L, *p* = 0.694), Δ CK (217 vs. 197 U/L, *p* = 0.652), Δ CRP (2.7 vs. 3.4 mg/L, *p* = 0.475), Δ bilirubin (2.4 vs. 2.8 µmol/L, *p* = 0.842), Δ creatinine (3.3 vs. 9.0 µmol/L, *p* = 0.085).

**Conclusion:**

BiB-PFA PVI provokes systemic responses involving inflammation, myocardial injury, and hemolysis. Adjunctive PWI increases application number but does not further increase biomarker release, supporting the biological safety of PWI.

## Graphical Abstract

**Figure:**
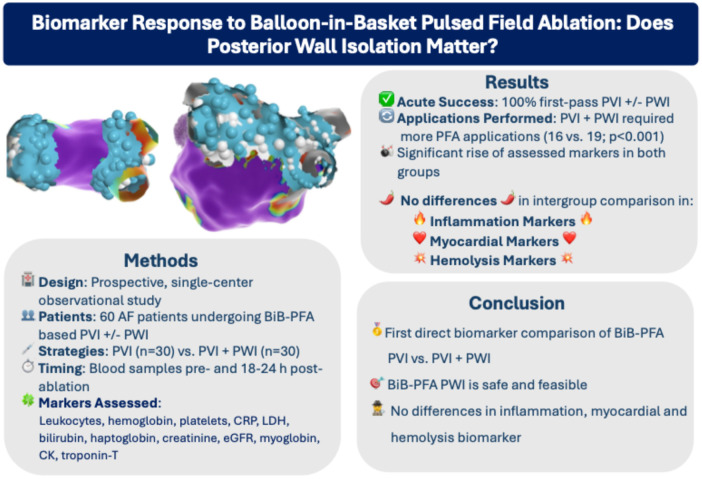

## Highlights

- First study to characterize systemic inflammatory, hemolytic, and myocardial biomarker responses after balloon-in-basket pulsed field ablation (BiB-PFA) with and without posterior wall isolation (PWI).
- Extending BiB-PFA from PVI to additional PWI did not increase inflammatory, myocardial, or hemolytic responses.
- Selective spline activation and localized energy delivery likely contribute to the favorable systemic safety profile of BiB-PFA, even during more extensive ablation strategies.

## Introduction

Pulsed field ablation (PFA)–based pulmonary vein isolation (PVI) has recently emerged as a transformative approach for the treatment of atrial fibrillation (AF), offering tissue selectivity for cardiomyocytes while largely sparing adjacent extracardiac structures such as the esophagus and phrenic nerve. This myocardial specificity aims to improve safety as thermal ablation may cause harmful complications such as esophageal injury or atrio-esophageal fistula formation (1). A novel balloon-in-basket (BiB)-PFA-system has further advanced PFA by enabling single-shot circumferential PVI with integrated mapping and energy delivery, allowing for efficient procedures with high acute isolation rates (2). In patients with persistent AF or posterior wall substrate, additional posterior wall isolation (PWI) is often performed to improve rhythm outcomes (3).

BiB-PFA PVI is known to induce systemic inflammation, myocardial injury, and potentially hemolysis (4–8). These markers provide insights into lesion characteristics, collateral tissue involvement, and procedure-related stress. However, the systemic impact of extending ablation beyond PVI including PWI using the BiB-PFA system remains unclear. To date, no studies have systematically compared post-procedural systemic biomarker responses between BiB-PFA PVI alone and PVI plus PWI.

The present study therefore aimed to analyze hemolytic, inflammatory, and myocardial injury biomarker responses after BiB-PFA in patients undergoing PVI alone vs. combined PVI plus PWI.

## Methods

### Study population and trial design

This prospective, single-center, observational study investigated systemic biomarker changes following BiB-PFA. Consecutive patients with AF undergoing first-time BiB-PFA were included and grouped as PVI only or PVI plus PWI. Venous blood samples were obtained before and 16–18 h after ablation. Seventeen patients of the PVI group were enrolled in the VOLT™ CE study, and 20 patients of the PVI + PWI group participated in the VOLT™ CE extended study (2).

Eligible participants were ≥18 years of age with documented AF and provided written informed consent. Exclusion criteria included active infection, autoimmune or chronic inflammatory conditions, recent myocardial infarction, neuromuscular disorders, or significant hepatic impairment to reduce potential biomarker confounders.

The study was approved by the local ethics committee (Lübeck Ablation Registry, approval number WF-028/15) and conducted in accordance with the Declaration of Helsinki (9).

### General procedural management

All patients underwent a standardized preprocedural workup according to institutional guidelines. In cases of elevated thromboembolic risk, transesophageal echocardiography was performed to exclude left atrial thrombus.

Vitamin K antagonists were continued at therapeutic INR levels (2.0–3.0), whereas direct oral anticoagulants were paused on the morning of the intervention.

Ablation procedures were conducted under deep sedation with propofol, midazolam, and fentanyl. In selected cases, continuous propofol was omitted to maintain partial responsiveness. For those patients, a multimodal analgesic approach including metamizole, midazolam, fentanyl, and lidocaine was used (10).

Femoral venous access was established via two ultrasound-guided punctures using 8 Fr sheaths. A diagnostic catheter was advanced into the coronary sinus (CS). Transseptal access was obtained under fluoroscopic guidance using a modified Brockenbrough technique.

Following transseptal puncture, intravenous unfractionated heparin was administered to maintain an activated clotting time above 300 s. Left atrial access was established via an SL1 sheath (Abbott).

In first 17 patients of the PVI group, procedures were performed according to the VOLT™ CE Mark protocol, including left atrial voltage mapping using a high-density mapping catheter (Advisor™ HD Grid, Abbott). In subsequent cases, anatomical reconstruction was conducted with the BiB-PFA-catheter alone. Following mapping, the transseptal sheath was exchanged for a steerable 13 Fr sheath (Agilis™ NxT, Abbott), and PV access was obtained using SL1 wire (Abbott).

PFA was delivered at 1,800 V with at least two rotated applications per vein. A maximum of eight applications per vein was permitted. For right-sided PVs, phrenic nerve capture was assessed using spline pacing. If diaphragmatic stimulation was observed, ablation was continued at 1,400 V with a minimum of three applications. Beyond study enrollment considerations, the decision to perform PVI alone or combined PVI + PWI was guided by procedural findings. In cases where posterior wall fibrosis was identified during left atrial mapping, additional PWI was performed at 1,800 V using selective spline activation to ensure direct wall contact, with a minimum of two applications. Isolation of all PVs and the posterior wall was confirmed by remapping with the BiB-PFA-catheter catheter at the end of the procedure in all patients.

In 20 patients of the PVI + PWI group, ablation followed the VOLT™ CE Mark extended protocol. Phrenic nerve capture was first confirmed via a CS catheter. After CS placement, left atrial mapping was performed using the BiB-PFA-catheter. Following a 20 min waiting period, remapping with the BiB-PFA-catheter catheter and repeat phrenic nerve testing were performed to confirm durable isolation and phrenic integrity.

### Postprocedural management

Hemostasis was achieved using either vascular closure devices or figure-of-eight sutures combined with a compression bandage. The bandage was removed after 1–4 h, and sutures were taken out on the following day. Transthoracic echocardiography was routinely performed immediately after the procedure, at one hour, and on the first postoperative day to exclude pericardial effusion. Oral anticoagulation was resumed six hours after ablation and continued for a minimum of two months. Long-term anticoagulation was guided by individual thromboembolic risk based on the CHA_2_DS_2_-VASc or CHA_2_DS_2_-VA score, in accordance with current guideline recommendations (11, 12). A class IC or III antiarrhythmic drug and a proton pump inhibitor was prescribed for two months.

### Intravenous fluid management

Intravenous fluids were administered during the procedure in patients with low blood pressure or clinical signs of hypovolemia. In such cases, up to 500–1,000 mL of balanced full-electrolyte solution were infused to maintain hemodynamic stability.

Post-procedurally, no standardized fluid regimen was applied. However, patients with baseline eGFR <60 mL/min/1.73 m^2^ received a single infusion of 500–1,000 mL balanced full-electrolyte solution on the first post-procedural day after routine blood sampling. Fluid administration was guided by clinical judgment, renal function, and individual volume status.

### Blood sampling and analysis

Venous blood samples were collected at two predefined time points: (1) prior to ablation, after femoral venous access, obtained directly from the venous sheath, and (2) on the morning of the first post-procedural day, obtained by direct peripheral venipuncture. All patients were fasting. A comprehensive biomarker panel was analyzed, including inflammatory (leukocytes, platelets, CRP), myocardial (troponin T, CK, myoglobin), renal (creatinine, eGFR), and hemolysis-related markers (LDH, total bilirubin, haptoglobin, Hb). Leukocytes and platelets were measured from EDTA whole blood (XN-9000, Sysmex), CRP by immunoturbidimetry (Cobas c503), troponin T and myoglobin by ECLIA (Cobas e801), CK by enzymatic UV photometry (Cobas c702), and LDH, bilirubin, haptoglobin, and creatinine by standard photometric assays (Cobas c503). eGFR was calculated using the CKD-EPI formula. All analyses were performed at the routine central laboratory of University Hospital Schleswig-Holstein, Campus Lübeck.

### Statistical analysis

Continuous data were tested for normality using the Shapiro–Wilk test and are presented as mean ± SD or median (Q1, Q3), as appropriate. Between-group comparisons were made using independent-samples *t*-tests or Mann–Whitney *U* tests. Within-group comparisons used paired *t*-tests or Wilcoxon signed-rank tests.

Correlations between biomarker changes and PFA applications were analyzed using Pearson or Spearman coefficients, based on distribution. Categorical variables are reported as absolute and relative frequencies and compared using Fisher's exact or Chi-square tests, depending on sample size.

Linear regression was performed for each Δ-biomarker to adjust for sedation regimen (conscious vs. deep) as well as study inclusion (VOLT™ CE Mark and VOLT™ CE Mark Extended) with results reported as β-coefficients and *p*-values.

All analyses were performed with IBM SPSS Statistics v29.0.1.0 (IBM Corp., Armonk, NY, USA). Two-sided *p*-values < 0.05 were considered statistically significant.

## Results

### Baseline characteristics

**Figure 1:**
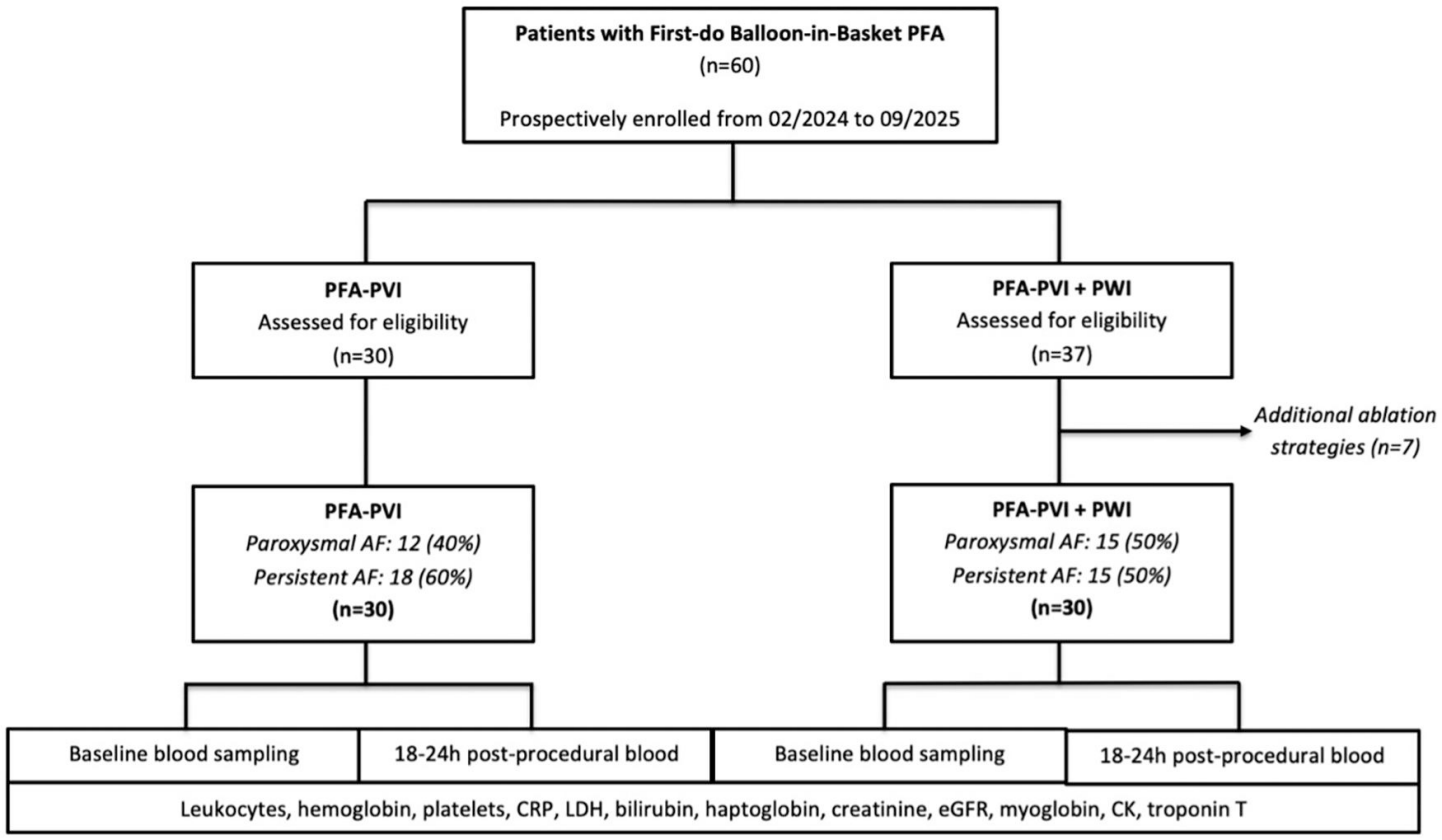
Study PRISMA (STROBE format). Represents the study flow chart. AF, atrial fibrillation; CK, creatine kinase; CRP, C-reactive protein; eGFR, estimated glomerular filtration rate; LDH, Lactate dehydrogenase; PFA, pulsed field ablation; PVI, pulmonary vein isolation; PWI, posterior wall isolation.

A total of 60 patients were included (30 PVI, 30 PVI + PWI). Median age was 67.5 years, and 40% were female. The proportion of patients with persistent AF was similar between groups (60% vs. 50%; *p* = 0.436). Other baseline demographics, comorbidities, and echocardiographic parameters were comparable (Table 1, Figure 1).

**Table 1:** Baseline characteristics of the study population. Values are presented as mean ± standard deviation, median (Q1; Q3) or number (percentage), as appropriate.

Variable	Total (*n* = 60)	PVI (*n* = 30)	PVI + PWI (*n* = 30)	*p*-value
Age (years)	67.5 (61.0; 75.0)	65.5 (60.3; 73.0)	69.0 (64.3; 75.8)	0.198
Sex (female), *n* (%)	24 (40%)	12 (40%)	12 (40%)	1
BMI (kg/m^2^)	27.3 (24.1; 29.3)	26.3 (23.9; 28.6)	27.6 (25.4; 29.4)	0.355
AF Type, *n* (%):				0.436
- Paroxysmal	27 (45%)	12 (40%)	15 (50%)	
- Persistent	33 (55%)	18 (60%)	15 (50%)	
Arterial hypertension, *n* (%)	38 (63.3%)	16 (53%)	22 (73.3%)	0.108
Diabetes mellitus, *n* (%)	10 (16.6%)	4 (13.3%)	6 (20%)	0.472
Coronary artery disease, *n* (%)	12 (20%)	6 (20%)	6 (20%)	1
Heart failure, *n* (%)	12 (20%)	4 (13.3%)	8 (26.6%)	0.197
Stroke/TIA, *n* (%)	7 (11.6%)	4 (13.3%)	3 (10%)	1
OSAS, *n* (%)	1 (1.6%)	0 (0%)	1 (3.3%)	1
CHA_2_DS_2_-VASc Score	2.0 (1.8; 4.0)	2.0 (1.0; 4.0)	2.5 (2.0; 4.0)	0.260
LAVI (mL/m^2^)	40.5 ± 13.8	39.9 ± 16.4	41.5 ± 9.8	0.847
LVEF (%)	55.0 (54.0; 57.0)	56.5 (55.0; 60)	55.0 (52.0; 55.0)	0.003
OAK	53 (88.3%)	24 (80.0%)	29 (96.6%)	0.044
Class I/III AAD at baseline, *n* (%)	27 (35%)	14 (27.5%)	13 (43.3%)	0.176
AF at procedure	30 (50%)	17 (56.6%)	13 (43.3%)	0.887
NTproBNP (ng/L)	434 (144; 878)	381 (86.5; 1,404)	451 (188; 617)	0.791

AF, atrial fibrillation; AAD, antiarrhythmic drug; BMI, body mass index; LAVI, left atrial volume index; LVEF, left ventricular ejection fraction; OAC, oral anticoagulation; OSAS, obstructive sleep apnea syndrome; PVI, pulmonary vein isolation; PWI, posterior wall isolation; TIA, transient ischemic attack.

### Procedural characteristics

Acute PVI was achieved in all patients (Table 2). The median number of energy applications was higher in the PVI + PWI group [19.0 (16.3; 21.0)] compared with PVI only [16.0 (14.0; 16.8); *p* < 0.001]. Procedure duration, LA-dwell and fluoroscopy time were comparable between groups. During PWI, a median of 3 (3; 4) splines were deselected. No procedural complications occurred.

**Table 2:** Procedural characteristics of the study population. Values are presented as mean ± standard deviation, median (Q1; Q3) or number (percentage), as appropriate.

Variable	Total (*n* = 60)	PVI (*n* = 30)	PVI + PWI (*n* = 30)	*p*-value
Procedure time (min)	60.7 ± 18.0	63.3 ± 15.5	58.0 ± 20.0	0.263
Conscious sedation (%)	25 (41.6)	5 (16.6)	25 (66.6)	<0.001
Fluoroscopy time (min)	8.4 (6.2; 10.6)	8.6 (6.3; 10.5)	7.4 (6.2; 10.6)	0.883
Dose area product (cGy·cm^2^)	362.5 (247.7; 559.8)	341.0 (257.0; 500.0)	400.5 (227.5; 595.8)	0.667
Left atrial dwell time (min)	46.78 ± 18.2	49.7 ± 18.3	43.8 ± 18.0	0.221
Number of cardioversions, *n*	0 (0; 1)	0 (0; 1)	0 (0; 1)	0.845
Contrast amount (mL)	40 (40.0; 50.0)	50 (40; 50)	40 (30; 40)	0.003
Successful acute PVI, *n* (%)	30 (100)	40 (100)	30 (100)	1
First-pass PVI, *n*(%)	30 (100)	30 (100)	30 (100)	1
PFA applications per subject, *n*	16.5 (15.0; 20)	16.0 (14.0; 16.8)	19.0 (16.3; 21.0)	<0.001
PFA applications LSPV *n*	4 (3; 4)	4 (4; 4)	4 (3; 4)	0.157
PFA applications LIPV, *n*	4 (3; 4)	4 (4; 4)	4 (3; 4)	0.096
PFA applications RSPV, *n*	4 (2.8; 4)	4 (3; 4)	4 (3; 4)	0.968
PFA applications RIPV, *n*	4 (3; 4)	4 (4; 4)	4 (3; 4)	0.352
PFA applications PW, *n*			4.5 (4; 5)	
Amount deselected splines PW, *n*			3 (3; 4)	
Major adverse events	0 (0)	0 (0)	0 (0)	1

LIPV, left inferior pulmonary vein; LSPV, left superior pulmonary vein; PFA, pulsed field ablation; PVI, pulmonary vein isolation; PW, posterior wall; PWI, posterior wall isolation; RIPV, right inferior pulmonary vein; RSPV, right superior pulmonary vein.

### Biomarkers—pre/post comparisons

Leukocytes increased in both PVI and PVI + PWI (*p* < 0.001) (Table 3). Platelets decreased significantly in PVI (*p* = 0.033) but not in PVI + PWI (*p* = 0.293). Hemoglobin showed a small, non-significant reduction (PVI *p* = 0.051; PVI + PWI *p* = 0.600). CRP increased in both groups (*p* < 0.001). Troponin T, CK, LDH, and myoglobin rose in both cohorts (all *p* < 0.001). Bilirubin increased (*p* < 0.001), while haptoglobin decreased in PVI + PWI (*p* < 0.001) but not in PVI (*p* = 0.654). Creatinine increased in PVI + PWI (*p* < 0.001) but not in PVI (*p* = 0.173). eGFR declined in PVI (*p* = 0.045) and PVI + PWI (*p* < 0.001). However, no acute renal failure occurred in both groups.

**Table 3:** Comparison of pre- and post-procedural biomarkers.

Biomarker	Timing	Total (*n* = 60)	*p*-value	PVI (*n* = 30)	*p*-value	PVI + PWI (*n* = 30)	*p*-value
Leukocytes (*10^9^/L)	Pre	6.2 (5.3; 7.6)	<0.001	6.2 (5.3; 7.4)	<0.001	6.1 (5.5; 7.7)	<0.001
	Post	7.8 (6.5; 9.1)		7.9 (6.5; 9.0)		7.6 (6.0; 9.3)	
Platelets (*10^9^/L)	Pre	196.0 (162.5; 236.0)	0.184	195.5 (156.3; 233.8)	0.033	200.0 (165.5; 241.3)	0.293
	Post	191.5 (155.5; 238.5)		190.5 (151.8; 214.3)		196.5 (166.3; 240.5)	
Hemoglobin (g/dL)	Pre	13.7 (12.6; 14.5)	0.322	14.0 (13.0; 14.7)	0.051	13.0 (12.3; 14.5)	0.600
	Post	13.5 (12.5; 14.2)		13.5 (12.7; 14.3)		13.1 (11.9; 14.2)	
CRP (mg/L)	Pre	1.2 (0.6; 2.0)	<0.001	1.1 (0.7; 1.7)	<0.001	1.2 (0.6; 2.5)	<0.001
	Post	5.7 (2.9; 9.4)		6.3 (3.1; 9.4)		5.4 (2.9; 8.3)	
Haptoglobulin (g/dL)	Pre	9.5 (7.5; 14.3)	0.998	9.6 (7.1; 13.9)	0.654	9.4 (8.0; 15.6)	<0.001
	Post	9.4 (6.8; 13.5)		9.8 (6.4; 13.8)		8.6 (6.9; 13.5)	
Bilirubin (µmol/L)	Pre	10.6 (7.7; 13.1)	<0.001	9.5 (7.2; 12.4)	<0.001	11.2 (8.0; 14.6)	<0.001
	Post	14.1 (10.2; 20.7)		12.3 (9.2; 15.5)		15.5 (11.4; 22.0)	
LDH (U/L)	Pre	184 (160; 202)	<0.001	189(163; 206)	<0.001	178(158; 197)	<0.001
	Post	241 (204; 264)		238 (222; 265)		244 (205; 263)	
Creatinine (µmol/L)	Pre	78.0 (62.8; 93.9)	<0.001	79.5 (70.0; 100.5)	0.173	76.8 (66.5; 89.0)	<0.001
	Post	85.0 (75.5; 98.6)		90.5 (75.3; 98.7)		83.2 (76.7; 98.2)	
eGFR (mL/min)	Pre	80.5 (62.8; 95.0)	<0.001	78.0 (62.5; 95.0)	0.045	83.5 (64.5; 94.8)	<0.001
	Post	77.0 (60.0; 91.0)		76.0 (64.5; 89.0)		78.0 (76.7; 98.2)	
Myoglobin (ng/mL)	Pre	44.0 (33.5; 60.0)	<0.001	42.0 (33.3; 57.5)	<0.001	44.0 (36.0; 62.0)	<0.001
	Post	56.0 (47.5; 79.0)		52.5 (45.5; 74.0)		63.0 (49.0; 80.0)	
CK (U/L)	Pre	84.0 (60.0; 132.0)	<0.001	85.0 (64.0; 134.3)	<0.001	81.0 (54.5; 117.0)	<0.001
	Post	320.5 (222.0; 443.5)		336.0 (228.0; 458.0)		314.0 (220.0; 427.0)	
Troponin-T (ng/L)	Pre	9.5 (7.1; 12.4)	<0.001	9.2 (6.6; 12.2	<0.001	9.5 (7.3; 12.2)	<0.001
	Post	1,102.0 (778.0; 1,663.0		1,123.0 (836.8; 1,571.3)		1,034.0 (778.0; 1,729.0)	

Values are presented as median (Q1; Q3).

CK, creatine kinase; CRP, C-reactive protein; eGFR, estimated glomerular filtration rate; LDH, lactate dehydrogenase; PVI, pulmonary vein isolation; PWI, posterior wall isolation.

### Intergroup comparison of delta values

**Figure 2:**
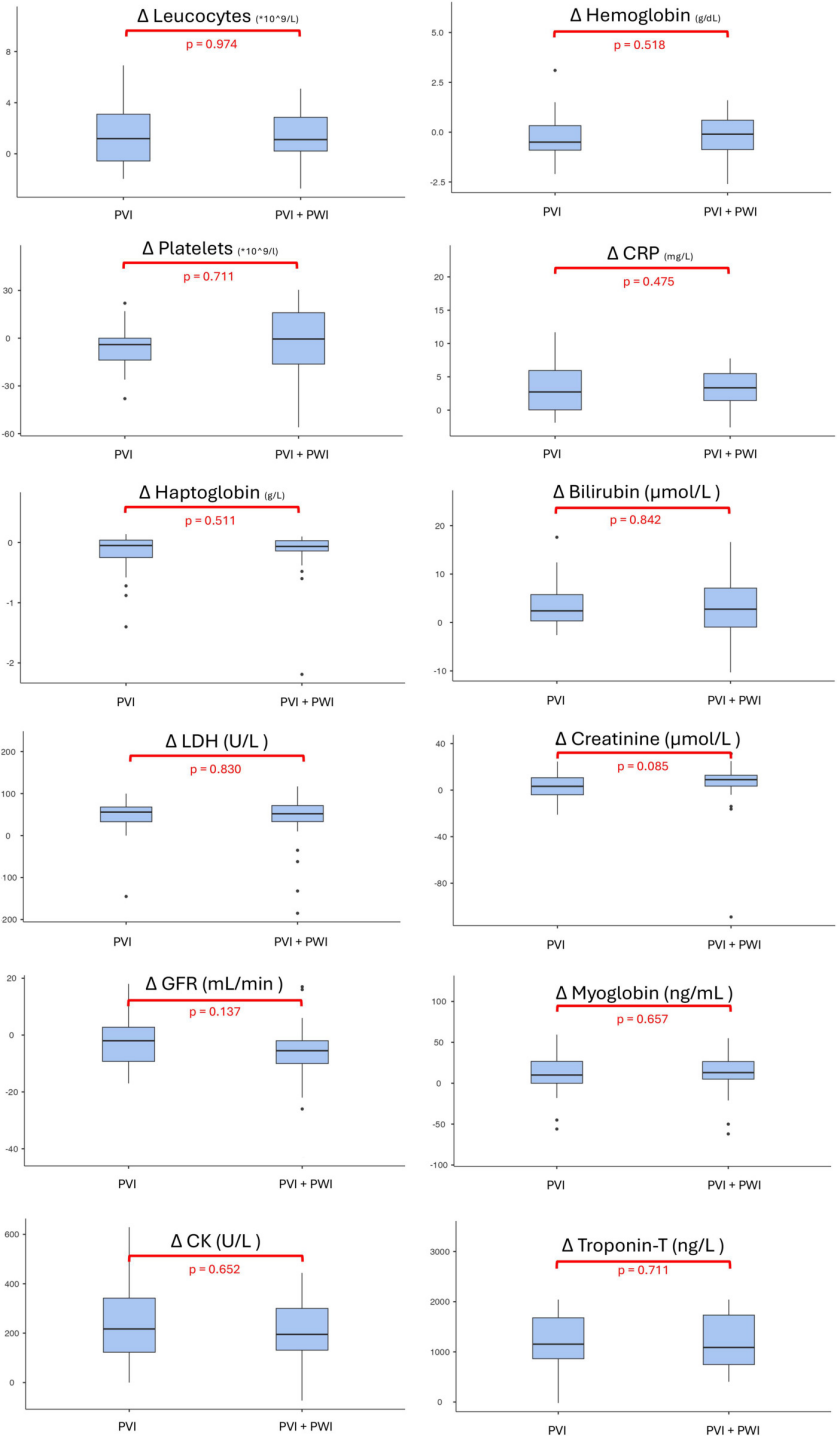
Comparison of delta values between the PVI group and the PVI + PWI group. Box plots represent the median (Q1; Q3) of changes from baseline to 18–24 h post-procedure. CK, creatine kinase; CRP, C-reactive protein; eGFR, estimated glomerular filtration rate; LDH, Lactate dehydrogenase; PVI, pulmonary vein isolation; PWI, posterior wall isolation.

No significant differences were observed between PVI and PVI + PWI regarding changes in leukocytes [1.2 (−0.6; 3.1) vs. 1.1 (0.2; 2.9) × 10^9^/L; *p* = 0.974], hemoglobin [−0.5 (−0.9; 0.3) vs. −0.1 (−0.9; 0.6) g/dL; *p* = 0.518], platelets [−4 (−13.8; 0) vs. −0.5 (−16.3; 16.0) × 10^9^/L; *p* = 0.711], CRP [2.7 (0.0; 5.9) vs. 3.4 (1.5; 5.5) mg/L; *p* = 0.475], haptoglobin [−0.3 (−1.6; 0.5) vs. −0.7 (−1.4; 0.3) g/L; *p* = 0.511], bilirubin [2.4 (0.3; 5.8) vs. 2.8 (−0.8; 7.9) µmol/L; *p* = 0.842], LDH [56.0 (33.0; 68.0) vs. 53.0 (34.0; 72.0) U/L; *p* = 0.830], creatinine [3.3 (−4.0; 10.7) vs. 9.0 (4.7; 13.2) µmol/L; *p* = 0.085], eGFR [−2.0 (−9.3; 2.8) vs. −5.0 (−10.0; −2.0) mL/min; *p* = 0.137], myoglobin [10.0 (0.0; 26.8) vs. 13.0 (5.0; 27.0) ng/mL; *p* = 0.657], CK [217.0 (122.8; 314.8) vs. 197.0 (147.0; 306.0) U/L; *p* = 0.652], and troponin T [1,153.5 (863.0; 1,679.8) vs. 1,029.0 (769.8; 1,720.0) ng/L; *p* = 0.694] (Figure 2, Table 4).

**Table 4:** Comparison of periprocedural changes (delta values) in biomarkers in total cohort.

Variable	Total (*n* = 60)	PVI (*n* = 30)	PVI + PWI (*n* = 30)	*p*-value
Δ Leucocytes (*10^9^/L)	1.2 (−0.1; 3.1)	1.2 (−0.6; 3.1)	1.1 (0.2; 2.9)	0.974
Δ Hemoglobin (g/dL)	−0.4 (−0.9; 0.6)	−0.5 (−0.9; 0.3)	−0.1 (−0.9; 0.6)	0.518
Δ Platelets (*10^9^/L)	−2.5 (−14.0; 10.0)	−4 (−13.8; 0)	−0.5 (−16.3; 16.0)	0.711
Δ CRP (mg/L)	3.1 (1.1; 5.7)	2.7 (0.0; 5.9)	3.4 (1.5; 5.5)	0.475
Δ Haptoglobulin (g/L)	−0.4 (−1.5; 0.5)	−0.3 (−1.6; 0.5)	−0.7 (−1.4; 0.3)	0.511
Δ Bilirubin (µmol/L)	2.4 (−0.2; 6.2)	2.4 (0.3; 5.8)	2.8 (−0.8; 7.9)	0.842
Δ LDH (U/L)	54.5 (32.8; 70.5)	56.0 (33.0; 68.0)	53.0 (34.0; 72.0)	0.830
Δ Creatinine (µmol/L)	6.3 (−0.8; 12.3)	3.3 (−4.0; 10.7)	9.0 (4.7; 13.20	0.085
Δ GFR (mL/min)	−4.0 (−10.0; 0.5)	−2.0 (−9.3; 2.8)	−5.0 (−10.0; −2.0)	0.137
Δ Myoglobin (ng/mL)	12.0 (0.0; 27.0	10.0 (0.0; 26.8)	13.0 (5.0; 27.0)	0.657
Δ CK (U/L)	210 (125.8; 318.8)	217.0 (122.8; 314.8)	197.0 (147.0; 306.0)	0.652
Δ Troponin T (ng/L)	1,129.0 (739.0; 1,715.7)	1,153.5 (863.0; 1,679.8)	1,029.0 (769.8; 1,720.0)	0.694

Values are presented as median (Q1; Q3).

Delta = post-procedural minus pre-procedural value.

CK, creatine kinase; CRP, C-reactive protein; eGFR, estimated glomerular filtration rate; LDH, lactate dehydrogenase; PVI, pulmonary vein isolation; PWI, posterior wall isolation.

In linear regression analyses adjusted for sedation regimen (conscious vs. deep) and study inclusion (VOLT™ CE Mark and VOLT™ CE Mark Extended), ablation strategy (PVI vs. PVI + PWI) was not independently associated with changes in leukocytes (*β* = 0.29, *p* = 0.700), hemoglobin (*β* = 0.23, *p* = 0.594), platelets (*β* = 3.22, *p* = 0.624), CRP (*β* = 0.38, *p* = 0.813), haptoglobin (*β* = 0.02, *p* = 0.875), total bilirubin (*β* = 0.81, *p* = 0.687), LDH (*β* = 3.40, *p* = 0.870), creatinine (*β* = −6.98, *p* = 0.209), eGFR (*β* = −2.34, *p* = 0.464), myoglobin (*β* = 9.44, *p* = 0.383), CK (*β* = −49.6, *p* = 0.223), or troponin T (*β* = −204, *p* = 0.360).

### Correlation between energy applications and biomarker changes

No significant correlations were observed between the number of applications and biomarker changes. LDH showed a non-significant trend (*p* = 0.055, Table 5).

**Table 5:** Correlation between energy applications and biomarker changes.

Marker	PVI and PVI + PWI (*n* = 60)	*p*-value
Leucocytes	−0.053	0.578
Hemoglobin	−0.039	0.671
Platelets	0.042	0.647
CRP	−0.079	0.409
Haptoglobin	−0.059	0.527
Billirubin	0.037	0.685
LDH	0.177	0.055
Creatinine	−0.009	0.923
GFR	−0.005	0.959
Myoglobin	−0.070	0.447
CK	0.030	0.743
Troponin-T	0.039	0.679

CK, creatine kinase; CRP, C-reactive protein; eGFR, estimated glomerular filtration rate; LDH, lactate dehydrogenase; PVI, pulmonary vein isolation; PWI, posterior wall isolation.

## Discussion

This prospective study investigated systemic biomarker responses following BiB-PFA for AF, comparing PVI only and combined PVI + PWI approaches. The main findings were:

- In both groups, inflammatory, myocardial, and hemolysis-related markers changed significantly after ablation.
- The median number of energy applications was higher in the PVI + PWI group as compared to the PVI only group.
- The magnitude of biomarker changes did not differ between PVI and PVI + PWI.
- BiB-PFA for PVI and adjunctive PWI was safe and feasible, with no procedural complications.

The present study provides novel insights into the systemic biological response to BiB-PFA for AF, comparing procedures limited to PVI with those including additional PWI. While BiB-PFA is increasingly recognized for its myocardial selectivity and favorable safety profile (2), data on extra-cardiac and systemic effects remain limited. Our results confirm that BiB-PFA, whether limited to PVI or extended to PWI, is safe and feasible, eliciting a consistent pattern of inflammatory, hemolytic, and myocardial biomarker changes without evidence of clinical apparent systemic or organ-specific injury.

### Inflammatory and myocardial biomarker response

In both groups, markers of inflammation and myocardial injury increased after ablation. Elevations in leukocytes and CRP likely reflect a short-term inflammatory response triggered by tissue injury and post-procedural physiological stress. Similar patterns have been observed after thermal ablation modalities such as radiofrequency or cryoballoon, but the magnitude of the inflammatory response in the current cohort appears modest in comparison, suggesting that BiB-PFA elicits less collateral tissue damage (4, 13, 14). The comparable rise in CRP between PVI and PWI indicates that extending lesion sets to the posterior wall does not amplify systemic inflammation. Biomarker responses thus appear to reflect the degree of extra-myocardial involvement and systemic activation rather than the absolute extent of ablated tissue, emphasizing the contained biological profile of PFA.

Troponin T and CK increased substantially in both groups, reflecting expected myocardial release due to targeted lesion formation. These findings are consistent with previous studies showing proportional troponin elevation after successful BiB-PFA despite its non-thermal nature (4). Importantly, similar magnitudes of myocardial marker release between PVI and PWI suggest that additional posterior wall applications do not translate into excessive myocardial injury, supporting the procedural safety of extended BiB-PFA lesion sets. This contained myocardial response may be attributable to the BiB-PFA-catheter's ability to maintain stable wall contact and deliver energy in a highly localized fashion, limiting current dispersion and minimizing collateral myocardial or pericardial involvement even during more extensive ablation strategies. Additionally, the posterior left atrial wall is anatomically thin, which may further contribute to the absence of a significant incremental troponin or CK rise—although this remains speculative and warrants confirmation in dedicated mechanistic studies.

### Hemolysis and renal response

Mild elevations in myoglobin and LDH were observed after ablation, most likely reflecting limited myocardial and skeletal muscle involvement related to instrumentation and sedation rather than true hemolysis. Hemolysis-related parameters, including bilirubin and haptoglobin, changed within expected physiological ranges and without evidence of systemic hemolysis. The modest reduction in haptoglobin and the minor decrease in eGFR observed in the PVI and PWI group reached statistical significance but lacked clinical relevance. These findings are in line with previous studies of our working group (4–7). The absence of relevant hemolysis is likely attributable to the BiB-PFA-catheter design, which enables selective spline activation, ensures optimal wall contact, and minimizes the amount of energy dissipated into the surrounding blood pool. These design features are particularly advantageous during extended ablation strategies such as PWI, where targeted energy delivery and controlled contact reduce the risk of collateral tissue and blood exposure, further supporting the safety of BiB-PFA in more extensive lesion sets. Overall, BiB-PFA did not induce meaningful hemolytic or renal injury.

### Dose–response considerations

Correlation analyses showed no significant relationships between the number of energy applications and biomarker changes. Only LDH displayed a weak trend, indicating minimal mechanical or procedural influence. Overall, the biological response to BiB-PFA appeared independent of total energy delivery, even in the setting of extended ablation strategies such as PWI. In contrast, previous studies such as NEMESIS using pentaspline PFA-sysems demonstrated a dose-dependent biomarker response (15, 16), suggesting that the absence of correlation in the present analysis may be attributable to a combination of differences in catheter design and lesion geometry, as well as the limited sample size and exploratory nature of the present study.

### Clinical implications

The BiB-PFA system combines PFA with a catheter design that allows selective spline activation and real-time visualization of wall contact, enabling precise energy delivery and minimizing dispersion into the blood pool. These features likely contribute to the absence of significant hemolysis or systemic inflammation, especially in additional ablation strategies such as PWI. This design-specific control of energy application represents a key safety advantage and may distinguish BiB-PFA from other PFA systems.

### Limitations

This study has several limitations. It was conducted at a single high-volume center with a limited sample size, which may restrict generalizability and limit statistical power for subgroup analyses. No prior sample size or power calculation was performed, as reliable effect size estimates for biomarker responses associated with PWI using a BiB-PFA-system were not available at the time of study initiation. Second, biomarkers were assessed only once (18–24 h post-ablation), limiting insight into temporal dynamics such as onset, peak, and resolution. Third, no apoptosis-related biomarkers were evaluated, which could have provided additional mechanistic information regarding the electroporative injury process. Fifth, myocardial injury was inferred from serum markers without imaging confirmation. Sixth, procedural and analytical protocols were standardized, unrecognized confounders such as hydration status or intraprocedural hemodynamics may have influenced biomarker levels. Seventh, a substantial proportion of patients were enrolled in the VOLT™ CE Mark and VOLT™ CE Extended studies, potentially introducing protocol-related bias. Eighth, the stepwise clinical adoption of the BiB-PFA system, together with increasing operator experience over time and protocol-specific procedural requirements, may have influenced procedural characteristics and biomarker responses and should be considered when interpreting the results. In addition, differential leukocyte counts, including neutrophils and lymphocytes, were not available, precluding calculation of the neutrophil-to-lymphocyte ratio. Also, only total bilirubin was assessed without differentiation into direct and indirect fractions, which would be particularly relevant to confirm procedure-related hemolysis. Furthermore, blood samples were obtained from different venous sites, which may have influenced biomarker measurements. Finally, while the findings suggest biological safety and feasibility of BiB-PFA for PVI and PWI, the study was not powered to detect rare adverse events, and results should be confirmed in larger multicenter cohorts.

## Conclusion

BiB-PFA enables safe and effective lesion formation for both PVI and PWI without increasing systemic, renal, or hemolytic injury. The lack of additional biomarker release with extended lesion sets, together with the absence of procedural complications, supports the favorable biological safety profile of this technology. These findings support BiB-PFA as a versatile and well-tolerated option for additional AF ablation strategies requiring extended lesion sets. Further studies integrating imaging and longitudinal biomarker profiling are needed to clarify the clinical relevance.

## Data Availability

The raw data supporting the findings of this study are not publicly available and will not be shared due to ethical and/or data protection restrictions.
